# The safety, efficacy, and influencing factors of biodegradable versus metallic occluders in the treatment of migraine patients with patent foramen ovale

**DOI:** 10.3389/fneur.2025.1677536

**Published:** 2025-10-07

**Authors:** Ke Sun, Chenyu Yang, Tian Zhou, Qing Zhou, Junchao Tian, Bo Sheng, Yunfei Wang, Xuetao Zhu, Zeping Hu

**Affiliations:** ^1^Department of Cardiology, The First Affiliated Hospital of Anhui Medical University, Hefei, China; ^2^Department of Intensive Care Unit, The First Affiliated Hospital of Bengbu Medical University, Bengbu, China; ^3^Department of Cardiology, Hefei High-Tech Cardiovascular Hospital, Hefei, China

**Keywords:** migraine, patent foramen ovale, PFO closure, biodegradable PFO occluder, metallic PFO occluder

## Abstract

**Objective:**

Compare the efficacy and safety of biodegradable versus metallic occluders in the treatment of migraine patients with patent foramen ovale (PFO), and analyze the predictive factors for migraine attack relief.

**Method:**

A single-center retrospective study included 133 patients diagnosed with migraine with PFO in Hefei High-Tech Cardiovascular Hospital from January 2021 to June 2024; 56 cases were treated with biodegradable occluders, and 77 cases were treated with metallic occluders. Collect preoperative baseline data, use the Migraine Disability Assessment (MIDAS) Questionnaire and monthly attack days to assess symptoms, and evaluate efficacy at 1-year follow-up after surgery. Effectiveness is defined as complete elimination of migraine symptoms or a reduction of ≥ 50% in monthly attack days.

**Results:**

The postoperative MIDAS score (biodegradable group: 10.45 ± 9.19 vs. metallic group: 11.32 ± 9.62, *p* = 0.453) and the number of migraine attacks per month (biodegradable group: 2.09 ± 1.58 days vs. metallic group: 1.87 ± 1.43 days, *p* = 0.506) after surgery showed no statistically significant differences in both groups. The MIDAS scores and the number of migraine attacks per month both demonstrated statistically significant differences between the preoperative and postoperative periods in each group (*p* < 0.05). No significant complications occurred during the perioperative period. The number of mild adverse events was limited and could not be statistically analyzed. Multivariate analysis showed that preoperative MIDAS scores, the number of migraine attacks per month, right-to-left shunts (RLS) at rest, and Platelet Crit (PCT) were the same independent predictors of postoperative remission for both types of occluders; in addition, C-reactive protein (CRP) is only an independent predictor of remission in the metallic occluder group.

**Conclusion:**

PFO closure can markedly alleviate symptoms in migraine patients with PFO. Both biodegradable and metallic occluders exhibit comparable efficacy in symptom improvement and safety at the 12-month postoperative mark. Patients presenting with more severe baseline migraine, RLS at rest, and elevated PCT may derive greater benefit from PFO closure, whereas lower CRP levels may portend a more favorable prognosis for those with metallic occluders. Biodegradable occluders may become a better choice for specific populations (such as young patients and those allergic to nickel).

## Introduction

During the embryonic stage, before the circulatory system is established, the foramen ovale serves as a normal physiological passage between the right and left atria of the fetal heart. It is fundamental for the fetal left heart system to obtain oxygen and essential nutrients from the maternal umbilical vein blood. Typically, infants’ foramen ovale fuses around 2 months after birth. However, if the fusion fails after the age of 3, most cases cannot be physiologically closed to form patent foramen ovale (PFO) (1). Epidemiological studies have shown that 25% of people still fail to close their foramen ovale on its own after reaching adulthood (2). Some studies suggest that PFO is an anatomical condition (3); most patients have no obvious clinical symptoms, and the flow rate of PFO is usually small, so people believed that PFO would not cause serious clinical diseases early. But with the discovery of multiple studies, PFO may induce various clinical diseases, including cryptogenic stroke, decompression sickness in divers, and migraine (4, 5).

Migraine is a common neurovascular disease. Patients experience repeated moderate to severe headaches in their daily lives, and at the same time, they often have symptoms such as nausea and vomiting. Meanwhile, some patients find it hard to tolerate light and sound when they fall ill (6, 7). It is estimated that about 1 billion people are affected by it (8). Migraine is more common in people under 50 years old, seriously affecting the life, work, and study of middle-aged and young people. The lack of information on the relevant pathogenesis limits the effective treatment of migraine (9).

Although current treatment methods are effective for some patients, there are still many patients who suffer from the impact of headaches (10). Recent research has emphasized the importance of right-to-left shunts (RLS), especially vasoactive substances as well as venous blood and microemboli, bypassing the pulmonary circulation metabolism through PFO channels and becoming triggering factors for migraine after entering arterial blood. At the same time, some chemicals and hormones are not cleared by the pulmonary circulation metabolism and directly enter the blood–brain barrier, leading to migraine (11).

At present, percutaneous catheter occlusion of PFO has become the preferred treatment strategy for some patients due to its advantages, such as less trauma, rapid recovery, rare complications, economy, and safety. However, metallic occluders made of nickel-titanium metal inevitably suffer from nickel release, tissue erosion, and compression during treatment, leading to serious complications such as allergies, atrioventricular block, and even heart perforation (12, 13). Compared with traditional metallic occluders, the biodegradable occluder is composed of a double umbrella-shaped disc made of polydioxanone (PDO) monofilament that degrades relatively quickly and a barrier film made of poly-L-lactic acid (PLLA) fabric that degrades relatively slowly. PDO and PLLA can be completely degraded and excreted from the body. They do not cause sustained inflammation or toxic reactions, and the degradation avoids long-term compression of the metal disk, thereby reducing the risk of long-term complications such as instrument erosion of surrounding structures, metal allergies, or thrombosis (14–16).

At present, PFO closure, especially the use of biodegradable devices, may provide potential therapeutic benefits for migraine patients, which has important clinical exploration value (17). However, there is still limited research data on the effectiveness and long-term safety of biodegradable PFO occluders in improving postoperative migraine symptoms. This study aims to compare the differences in the impact of biodegradable occluders and metallic occluders on migraine symptoms in patients after PFO closure through preoperative standardized questionnaire surveys and postoperative systematic follow-up evaluations. The study will also analyze the differences in predictive factors for postoperative migraine improvement between the two materials of occluders in order to provide better options for clinical patient diagnosis and treatment strategies.

## Methods

### Participants

This study was a single-center, retrospective study. A total of 145 migraine patients diagnosed with PFO at Hefei High-tech Cardiovascular Hospital from January 2021 to June 2024 were selected. Migraine diagnosed according to the International Classification of Headache Disorders, 3rd edition (ICHD-3), excluding secondary migraine. Right heart contrast transthoracic echocardiography (cTTE) confirmed the presence of RLS at the foramen ovale, with a shunt volume of grade II or above, or transesophageal echocardiography (TEE) confirmed the presence of PFO. The patient and their family members have given consent and, the patient can cooperate with the surgery; they meet the indications for PFO closure and have an unsatisfactory response or cannot tolerate conventional drug therapy or acute treatment for migraine. Migraine patients have had an average of ≥ 2 headache days per month in the past 3 months, and have had no acute changes or fluctuations in migraine.

Exclusion criteria include no evidence of RLS or PFO excluded by TEE; secondary migraine caused by the following diseases (stroke, intracranial tumor, other types of headache) or excessive alcohol consumption or repeated drug use within the past year; contraindications for PFO closure (coagulation disorders, inferior vena cava or pelvic vein thrombosis, etc.); significant abnormalities in liver and kidney functions or a survival time of less than one year; concurrent angina pectoris, bleeding disorders, etc.; contraindications to aspirin or clopidogrel treatment; the patient is already pregnant or has plans to conceive in the near future.

### Data collection and variables

This study followed the principles of the Declaration of Helsinki and was approved by the Ethics Committee of Hefei High-tech Cardiovascular Hospital. All the selected cases had informed consent forms provided by the patients themselves or their authorized relatives (including surgical indications, surgical efficacy, surgical risks, and surgery-related complications). Relevant clinical data were obtained through means such as computer, telephone, or face-to-face consultation, and structured questionnaires were established, and the medical histories of the patients were recorded. These data include the patient’s age, gender, body mass index, history of hypertension, history of hyperlipidemia, history of diabetes, smoking status, and size and type of the occluder. Relevant hematological indicators such as red blood cell count (RBC), white blood cell count (WBC), hemoglobin (HB), platelet count (PLT), mean platelet volume (MPV), platelet distribution width (PDW), platelet crit (PCT), C-reactive protein (CRP), D-dimer (DD), triglyceride (TG), glucose (GLU), low-density lipoprotein (LDL), etc. Relevant transthoracic echocardiography (TTE) indicators such as Left Ventricular Ejection Fraction‌ (LVEF), Left Atrial Diameter (LAD), Left Ventricular Diastolic Diameter (LVDD), left ventricular end-diastolic volume (LVDS), shunt grading of cTTE (grade II: moderate shunt, grade III and above: massive shunt), and whether there is RLS at rest, etc.

The Migraine Disability Assessment (MIDAS) is used to assess the frequency and severity of migraines in patients before and after surgery. According to the MIDAS scoring criteria, the severity of headaches is classified into four categories: little or no disability (0–5 points), mild disability (6–10 points), moderate disability (11–20 points), and severe disability (≥ 21 points). In summary, based on the MIDAS scoring scale, the severity of the impact of migraines on a patient’s work, study, social interaction, and family life activities within three months can be evaluated, as well as the patient’s perception of pain. Moreover, the number of days of migraine attacks each month in the three months before the operation and the 10–12 months after the operation can be recorded. All data were collected by other hospital staff.

### Echocardiography

#### TTE

The patient was placed in the left lateral position, exposing the anterior thoracic area. The probe was selected according to the examination requirements. Each section of the heart was scanned in the standard sequence, and hemodynamic assessment was performed using color Doppler technology.

#### cTTE

The patient was placed in the left lateral position. 8 mL of normal saline + 1 mL of air + 0.5–1 mL of autologous blood was taken and repeatedly and rapidly injected through a three-way tube for ≥20 times to form uniform microbubbles. Select the four-chamber pericardium section at the apex, clearly display the atrial septum, and rule out spontaneous angiography or thrombosis in the left heart. Rapid injection through the anterior cubital vein to activate microbubbles (initial dose ≤ 1 mL), a subsequent additional dose ≤ 5 mL, and repetition if necessary (interval > 5 min). Continuously record the sequence of microbubble appearance (right atrial - right ventricular - left cardiac system). Grade I: Left ventricular microbubbles 1–10 per frame; Grade II: 11–30 frames per frame; Grade III: > 30 frames per frame or left heart turbidity.

#### TEE

The patient was placed in the left lateral position, with the local anesthetic drug in the mouth for a moment. The ultrasound probe was slowly sent through the mouth into the patient’s esophagus. The atrial septum was continuously scanned at 15° intervals from the back to the front of the heart to observe the gap between the primary septum and the secondary septum and the presence of shunt signals. A positive result indicates the presence of PFO, while a negative result requires reference to other imaging examinations. Clarify the anatomical structure, size, etc. of the PFO to provide a reference for the selection of the occluder.

### PFO operation

**Figure 1:**
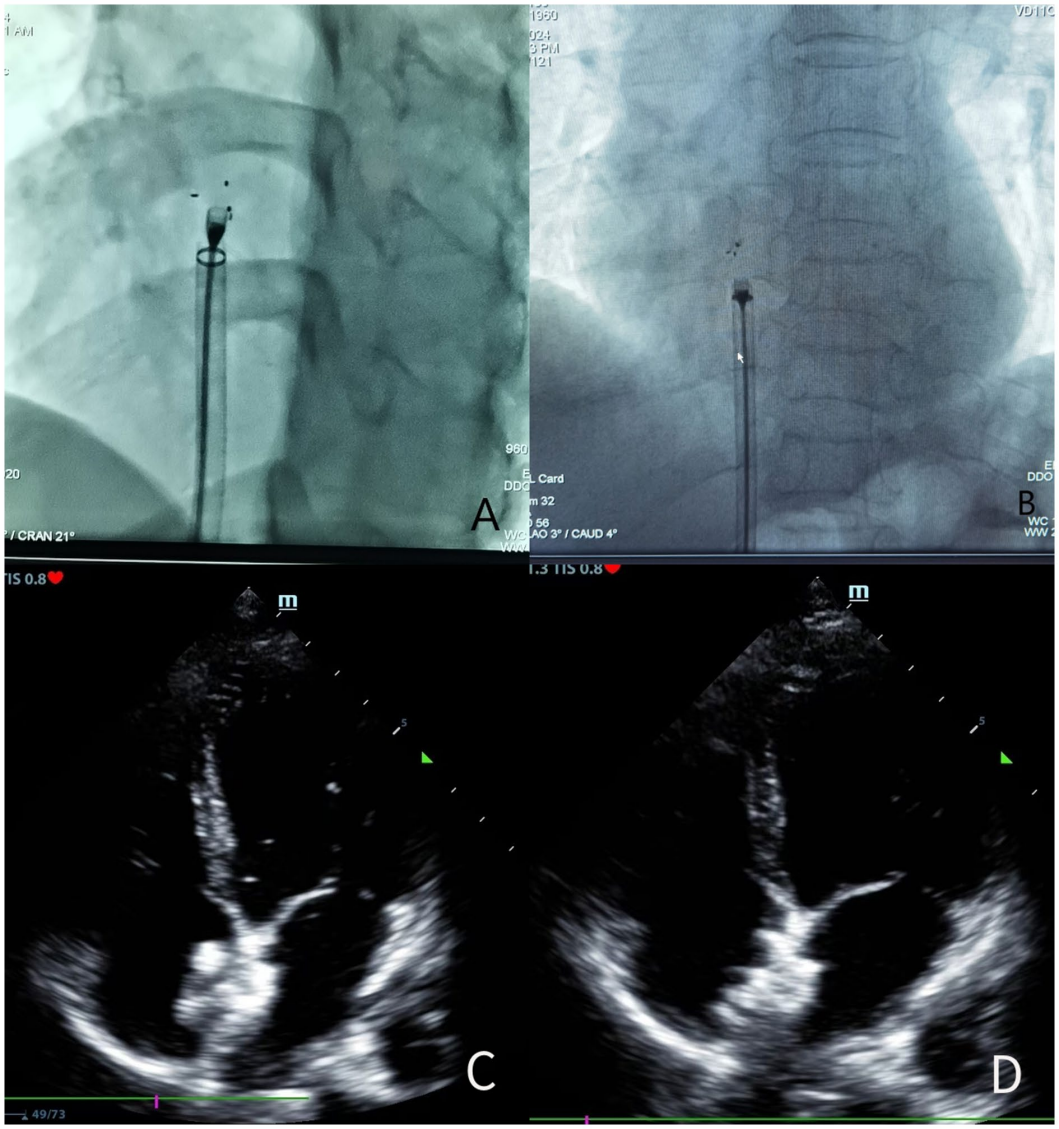
**(A)** Before the release of the biodegradable occluder under the X-ray line; **(B)** After the release of the biodegradable occluder under the X-ray line; **(C)** Before the release of the biodegradable occluder by TTE during the operation; **(D)** After the release of the biodegradable occluder by TTE during the operation.

Preventive antibiotics were administered intravenously 15 min before the operation. During the operation, the patient lay flat on the operating table. Lidocaine was used for local anesthesia of the right groin. The right femoral vein path was selected, and a 6F venous sheath was inserted. Heparin was administered according to the patient’s body weight (80–100 IU/kg) (18). It was replaced with an 8.5F Swartz sheath tube and sent down to the superior vena cava. The tip of the sheath tube pointed to the atrial septum. When there was a jump, an attempt was made to send the guide wire through the foramen ovale into the left pulmonary vein. Then, the hardened guide wire and the 10F-12F delivery sheath tube were inserted into the occluder. After releasing the left atrial front umbrella, the sheath tube was withdrawn; subsequently, the right atrial rear umbrella was released. After confirming that the occluder had no displacement, subsequent operations were carried out. For absorbable occluders, after confirming through ultrasound that the occluder is attached to the left atrial septum, the tail of the line is tightened to shape the occluder and make it adhere tightly to the atrial septum, and then subsequent operations are carried out (Figure 1). The delivery sheaths, metals, and absorbable occluders used during the operation were all purchased from Shanghai Shape Memory Alloy LTD or Beijing Huayi Shengjie LTD.

### PFO closure and postoperative follow-up

All patients used occluders to close the foramen ovale. All surgical patients were given a standard dose of low-molecular-weight heparin for 48 h after the operation and were treated with aspirin for 6 months and clopidogrel for 3 months. cTTE was performed at 1, 6, and 12 months after PFO closure. If the cTTE was negative, it was considered that there was no RLS 5 s after the re-injection. Under the Valsalva movement, the number of microbubble signals in the left atrium within five cardiac cycles was recorded. The absence of microbubbles is regarded as no residual shunt. 1 to 10 microbubbles per frame are regarded as mild residual shunt (Grade I); 11–30 microbubbles are regarded as moderate residual shunt (Grade II); more than 30 microbubbles per frame are regarded as severe residual shunt (Grade III). During the follow-up period, each patient records the MIDAS scores and the number of migraine attacks each month by phone or in the outpatient department within ± 5 days at each time point, whether there are any serious postoperative complications (such as the detachment or displacement of the occluder, significant pericardial effusion, new-onset arrhythmia, etc.). All data were collected by other hospital staff. The definition of migraine improvement after closure: 1. Complete elimination of symptoms; 2. >50% reduction in the number of migraine attacks per month (19). Effective closure and defined procedures resulting in no residual or mild residual shunt (20).

### Statistical analysis

The relevant data were analyzed and processed using SPSS 26.0 software. The counting data uses the number of cases and the percentage of cases of this case for statistics. For continuous variables whose measurement data conform to a normal distribution, the relevant results are expressed as x¯ ± s, and the independent sample t-test was used for comparison between groups. Perform the Wilcoxon rank sum test on continuous data with a non-normal distribution. The data comparison of categorical variables was conducted using the chi-square test. Univariate analysis was conducted on the indicators related to the improvement of postoperative migraine, and the indicators with *p* < 0.05 were included in the multivariate logistic regression analysis. The significance level *α* = 0.05, and *p* < 0.05 was considered statistically significant.

## Result

### Basic information of patients

A total of 145 patients were enrolled before the operation. 60 patients were included in the biodegradable occluder group, and 85 patients were included in the metallic occluder group. Among them, 1 patient in the biodegradable occluder group gave up the operation before the operation, and the follow-up of 3 patients was lost. In the metallic occluder group, 2 patients had pericardial tamponade during the operation, 1 patient gave up the operation due to the inability of the guidewire to pass, and the follow-up of 5 patients was lost. Therefore, a total of 133 patients successfully had the occluder implanted and completed the follow-up. Among them, biodegradable occluders were successfully implanted in 56 patients, and metallic occluders were successfully implanted in 77 patients. Table 1 shows the corresponding basic information of the enrolled patients. There were no significant differences between the two groups in terms of age, gender, BMI, hypertension, diabetes, smoking, echocardiography, and hematological indicators. The preoperative MIDAS score and the number of attack days per month were similar and matched (*p* > 0.05, Table 1).

**Table 1:** Patient characteristics.

Variable	Biodegradable occluder group *n* = 56	Metallic occluder group *n* = 77	*p*-value
Age (mean ± SD)	36.84 ± 11.01	40.90 ± 13.01	0.060
Sex (*n*, %)			0.261
Female Male	38 (67.9) 18 (32.1)	59 (76.6) 18 (23.4)	
BMI (kg/m^2^)	23.35 ± 4.26	22.48 ± 3.54	0.200
History of hypertension			0.418
Yes No	6 (10.7) 50 (89.3)	12 (15.6) 65 (84.4)	
History of diabetes			0.745
Yes No	2 (3.6) 54 (96.4)	2 (2.6) 75 (97.4)	
History of smoking			0.079
Yes No	11 (19.6) 45 (80.4)	7 (7.1) 70 (90.9)	
LVEF (%)	66.91 ± 4.87	66.06 ± 5.51	0.360
LAD (mm)	30.63 ± 4.16	31.66 ± 4.05	0.152
LVDS (mm)	29.43 ± 3.45	30.18 ± 2.91	0.174
LVDD (mm)	45.93 ± 4.04	47.12 ± 3.62	0.077
cTEE			0.067
Grade II Grade III and above	16 (28.6) 40 (71.4)	34 (44.2) 43 (55.8)	
RLS at rest			0.052
Yes No	21 (37.5) 35 (62.5)	17 (22.1) 60 (77.9)	
DD	0.41 ± 0.31	0.40 ± 0.35	0.762
WBC	5.72 ± 1.39	6.18 ± 1.93	0.131
RBC	4.54 ± 0.40	4.42 ± 0.44	0.112
HB	131.16 ± 14.19	130.35 ± 18.24	0.782
PLT	234.64 ± 59.31	220.29 ± 57.66	0.164
PCT	0.21 ± 0.05	0.20 ± 0.05	0.407
MPV	9.39 ± 1.22	9.54 ± 0.97	0.419
PDW	10.32 ± 2.18	10.22 ± 1.22	0.789
CRP	1.62 ± 4.20	1.18 ± 2.53	0.280
TG	1.64 ± 1.03	1.50 ± 1.15	0.454
GLu	5.32 ± 0.70	5.38 ± 1.07	0.750
LDL	2.78 ± 0.92	2.64 ± 0.63	0.311
Preoperative MIDAS	37.80 ± 7.30	40.05 ± 7.75	0.071
Postoperative MIDAS	10.45 ± 9.19	11.32 ± 9.62	0.453
Preoperative attack days (/m)	5.86 ± 1.54	5.92 ± 1.84	0.920
Postoperative attack days (/m)	2.09 ± 1.58	1.87 ± 1.43	0.506

LVEF, left ventricular ejection fraction‌; LAD, left atrial diameter; LVDS, left ventricular end-diastolic volume; LVDD, left ventricular diastolic diameter; cTEE, right heart contrast transthoracic echocardiography; RLS at rest, split right to left in the resting state; DD, D-dimer; WBC, white blood cell count; RBC, red blood cell count; HB, hemoglobin; PLT, platelet count; PCT, platelet hematocrit; MPV, mean platelet volume; PDW, platelet distribution width; CRP, C-reactive protein; TG, triglyceride; GLU, glucose; LDL, low-density lipoprotein.

### Results of transcatheter closure

During the implantation of PFO occluders in the two groups of patients, one patient in the biodegradable occluder group had intraoperative hypotension, and two patients were successfully implanted after atrial septal puncture (during the operation, the guide wire could not pass through the PFO. After communicating with the patient and their family, the patient’s family agreed and signed to confirm the atrial septal puncture). In the metallic occluder group, three patients had transient atrioventricular block during the operation and recovered within 24 h after the operation. The remaining patients underwent bedside electrocardiogram examination within 24 h post-operation, and no instances of atrial fibrillation, atrial flutter, atrioventricular block, or ventricular arrhythmia were detected. Two patients developed a small amount of pericardial effusion after the operation, and neither of them received any treatment. The pericardial effusion was spontaneously absorbed. One patient had a fever after the operation. No serious complications occurred, such as coronary artery air embolism, thrombosis on implanted devices, persistent arrhythmia, thromboembolism related to implanted devices, cardiac perforation, infective endocarditis, etc. The occluders used during the operation were all from Shanghai Shape Memory Alloy LTD or Beijing Huayi Shengjie LTD. The application of the biodegradable occluder group occluder is as follows: 18 mm (*n* = 4), 18–24 mm (*n* = 2), 24 mm (*n* = 29), 28 mm (*n* = 20), 34 mm (*n* = 1); metallic occluder group: 18 mm (*n* = 19), 18–24 mm (*n* = 7), 18–25 mm (*n* = 6), 24 mm (*n* = 21), 25 mm (*n* = 6), 28 mm (*n* = 10), 30 mm (*n* = 7), 34 mm (*n* = 1).

### Follow-up safety evaluation of PFO closure

All the followed patients did not receive any other drug or surgical treatment during this period. All patients received standardized antiplatelet therapy as required by the study, and no patient stopped taking the medicine on their own. During the follow-up period, all occluders were in place, and no serious complications occurred. The two groups of patients reported minor adverse events within one month after the operation. In the biodegradable occluder group, there were 8 cases of palpitations and 3 cases of chest discomfort. In the metallic occluder group, there were 12 cases of palpitations, 5 cases of chest discomfort, 2 cases of weakness, and 1 case of breathing difficulties. In the cases reported with palpitations, electrocardiogram (ECG) examinations were performed, and no significant abnormalities were found in these cases. At the 6-month follow-up examination, the adverse event-related symptoms of most patients subsided.

### Residual shunt evaluation

**Figure 2:**
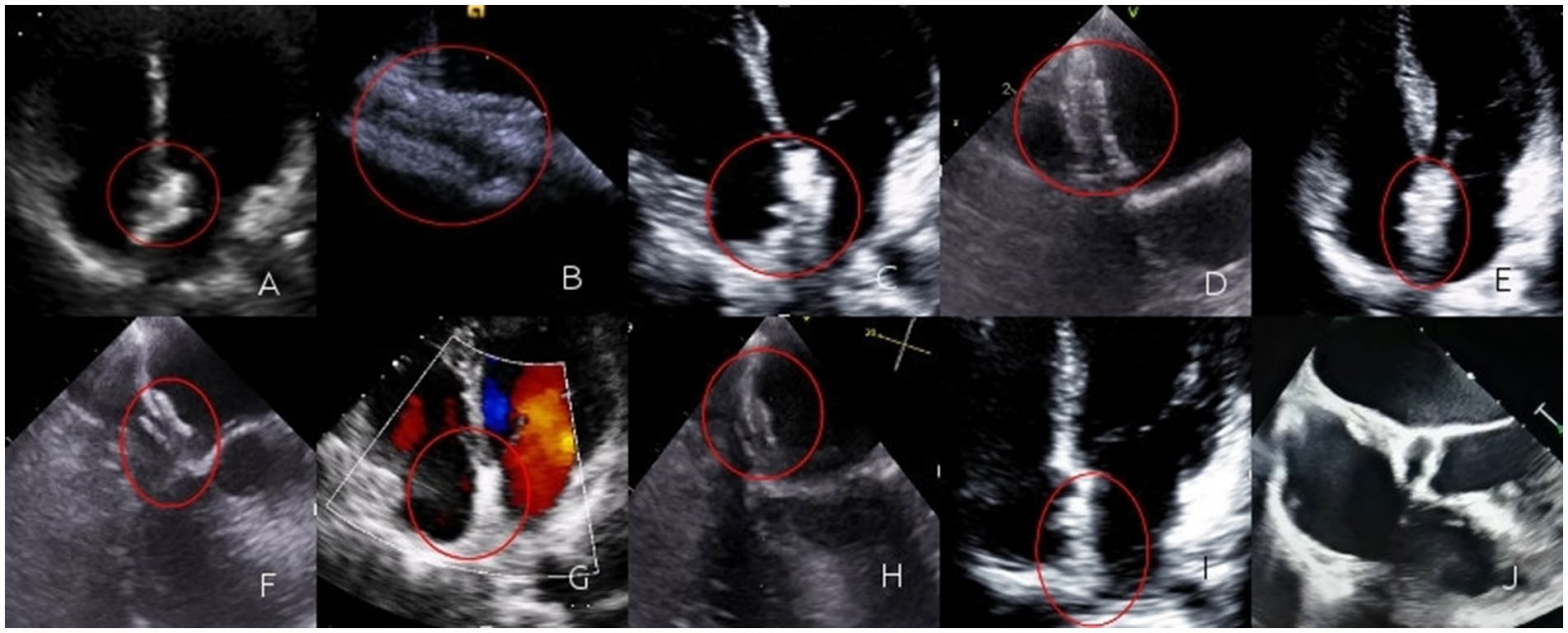
**(A)** Immediately after the operation, TTE revealed three layers of interlayers in the contour of the occluder. The occluder was highlighted and moved synchronously with the atrial septum; **(B)** Immediately after the operation, a circular contour of the left disc could be seen by TEE; **(C)** One month after the operation, TTE revealed three layers of interlayers in the contour of the occluder. The occluder was highlighted and moved synchronously with the atrial septum; **(D)** One month after the operation, a circular contour of the left disc could be seen by TEE; **(E)** Three months after the operation, TTE revealed three layers of interlayers in the contour of the occluder. The occluder was highlighted and moved synchronously with the atrial septum; **(F)** Three months after the operation, a circular contour of the left disc could be seen by TEE; **(G)** Six months after the operation, TTE revealed the occlude was highlighted, the contour of the occluder was blurred, the three-layer dissection structure gradually became blurred, and it moved synchronously with the atrial septum; **(H)** Six months after the operation, a circular contour of the left disc could be seen by TEE, the occluder gradually degrades; **(I)** One year after the operation, the TTE was not clear enough, and the three layers of interlayers could not be observed; **(J)** One year after the operation, TEE revealed regenerated tissue in the form of the occluder. The regenerated tissue faintly formed a three-layer interlayer with the atrial septum.

During the 1-month follow-up, 52 patients in the biodegradable occluder group rechecked cTTE. Among them, 8 patients had a grade I shunt, 4 patients had a grade II shunt, and no obvious shunt was observed in the rest. In the metallic occluder group, 70 patients rechecked cTTE. Among them, 13 patients had a grade I shunt, 8 patients had a grade II shunt, and no obvious shunt was observed in the rest. Rates of residual shunt were similar between two groups (7.7% vs. 11.4%; *p* = 0.493). During the 6-month follow-up, 45 patients in the biodegradable occluder group rechecked cTTE. Among them, 8 patients had a grade I shunt, 3 patients had a grade II shunt, and no obvious shunt was observed in the rest. In the metallic occluder group, 63 patients rechecked cTTE. Among them, 10 patients had a grade I shunt, 7 patients had a grade II shunt, and no obvious shunt was observed in the rest. Rates of residual shunt were similar between two groups (6.7% vs. 11.1%; *p* = 0.432). During the 1-year follow-up period, 38 patients in the biodegradable occluder group had a recheck cTTE. Among them, 7 patients had a grade I shunt, 1 patient had a grade II shunt, and no obvious shunt was observed in the rest. In the metallic occluder group, 51 patients rechecked cTTE. Among them, 8 patients had a grade I shunt, 4 patients had a grade II shunt, and no obvious shunt was observed in the rest (Figure 2). Rates of residual shunt were similar between two groups (3.6% vs. 7.8%; *p* = 0.291).

### Comparison of migraine relief

Comparing the postoperative MIDAS scores (biodegradable occluder group: 10.45 ± 9.19 vs. metallic occluder group: 11.32 ± 9.62, *p* = 0.453) and the number of days of migraine attacks each month after the operation (biodegradable occluder group: 2.09 ± 1.58 days vs. metallic occluder group: 1.87 ± 1.43 days, *p* = 0.506) between the two groups, the differences were not statistically significant. In the biodegradable occluder group, the preoperative MIDAS scores were 37.80 ± 7.30, and the postoperative MIDAS scores were 10.45 ± 9.19, *p* < 0.05. The number of days of migraine attacks each month before the operation was 5.86 ± 1.54, and the number of days of migraine attacks each month after the operation was 2.09 ± 1.58, *p* < 0.05. In the metallic occluder group, the preoperative MIDAS scores were 40.05 ± 7.75, and the postoperative MIDAS scores were 11.32 ± 9.62, p < 0.05. The number of days of migraine attacks each month before the operation was 5.92 ± 1.84, and the number of days of migraine attacks each month after the operation was 1.87 ± 1.43, p < 0.05. The MIDAS scores before and after the operation and the number of days of migraine attacks each month in both groups reached statistical significance (Figure 1).

According to the definition of complete elimination of migraine or a reduction of ≥ 50% in monthly attack days as migraine improvement, the biodegradable occluder group and the metallic occluder group were, respectively, divided into the remission group and the non-remission group.

The biodegradable occluder group was divided into the remission group and the non-remission group according to whether migraine improved one year after surgery. By comparing the preoperative baseline data of the two groups, it was found that there were differences between the two groups in preoperative MIDAS scores, the number of attack days per month before surgery, shunt grading of cTTE, whether there was RLS at rest, and PCT. When the above indicators were included in the multivariate logistic regression analysis, it was found that preoperative MIDAS scores (*p* = 0.049), the number of attack days per month before surgery (*p* = 0.037), the presence of RLS at rest (*p* = 0.046), and PCT (*p* = 0.039) were independent risk factors for postoperative migraine relief in patients of the biodegradable occluder group (Tables 2, 3).

**Table 2:** Baseline data between the migraine remission and non-remission groups with biodegradable occluders.

Variables	Non-remission group *n* = 17	remission group *n* = 39	*p*-value
Age (mean ± SD)	33.71 ± 10.47	38.21 ± 11.07	0.161
Sex (*n*, %)			0.739
Female Male	11 (64.7) 6 (35.3)	27 (69.2) 12 (30.8)	
BMI (kg/m^2^)	24.38 ± 3.99	22.91 ± 4.35	0.239
History of hypertension			0.440
Yes No	1 (5.9) 16 (94.1)	5 (12.8) 34 (87.2)	
History of diabetes			0.342
Yes No	0 (0) 17 (100)	2 (5.1) 37 (94.9)	
History of smoking			0.629
Yes No	4 (23.5) 13 (76.5)	7 (17.9) 32 (82.1)	
LVEF (%)	68.35 ± 5.89	66.28 ± 4.28	0.145
LAD (mm)	30.94 ± 3.93	30.49 ± 4.30	0.711
LVDS (mm)	28.53 ± 3.61	29.82 ± 3.35	0.200
LVDD (mm)	46.06 ± 3.75	45.87 ± 4.21	0.875
cTEE			0.043
Grade II Grade III and above	8 (47.1) 9 (52.9)	8 (20.5) 31 (79.5)	
RLS at rest			0.009
Yes No	2 (11.8) 15 (88.2)	19 (48.7) 20 (51.3)	
DD	0.29 ± 0.33	0.46 ± 0.29	0.053
WBC	6.09 ± 1.45	5.56 ± 1.34	0.192
RBC	4.66 ± 0.34	4.49 ± 0.41	0.140
HB	133.82 ± 13.09	130.00 ± 14.65	0.359
PLT	246.18 ± 57.64	229.62 ± 60.05	0.341
PCT	0.16 ± 0.05	0.23 ± 0.04	<0.001
MPV	9.26 ± 1.05	9.44 ± 1.29	0.612
PDW	10.11 ± 1.61	10.41 ± 2.40	0.647
CRP	2.87 ± 7.34	1.07 ± 1.39	0.787
TG	1.55 ± 0.96	1.68 ± 1.07	0.675
GLU	5.27 ± 0.69	5.34 ± 0.71	0.734
LDL	2.59 ± 0.65	2.86 ± 1.02	0.327
Preoperative MIDAS	30.53 ± 4.35	40.97 ± 3.93	<0.001
Preoperative attack days (/m)	4.58 ± 0.87	6.41 ± 1.45	<0.001

LVEF, left ventricular ejection fraction‌; LAD, left atrial diameter; LVDS, left ventricular end-diastolic volume; LVDD, left ventricular diastolic diameter; cTEE, right heart contrast transthoracic echocardiography; RLS at rest, split right to left in the resting state; DD, D-dimer; WBC, white blood cell count; RBC, red blood cell count; HB, hemoglobin; PLT, platelet count; PCT, platelet hematocrit; MPV, mean platelet volume; PDW, platelet distribution width; CRP, C-reactive protein; TG, triglyceride; GLU, glucose; LDL, low-density lipoprotein.

**Table 3:** Multivariate analysis of the migraine remission and non-remission groups with biodegradable occluders.

Predictor	Coef	SE	Wald Z	*P* value	OR	95%CI
Preoperative MIDAS	0.302	0.153	3.885	0.049	1.352	1.002–1.83
Preoperative attack days	3.856	1.849	4.349	0.037	47.253	1.26–1770.57
Shunt grading of cTTE	1.418	1.663	0.727	0.394	4.128	0.159–107.45
RLS at rest	6.441	3.223	3.993	0.046	626.86	1.13–347450.82
PCT	50.528	24.418	4.282	0.039	8.79E+21	14.42–5.36E+42

RLS at rest, split right to left in the resting state; PCT, platelet hematocrit; Coef, coefficient; SE, standard error; OR, odds ratio; CI, confidence interval.

The metallic occluder group was divided into the remission group and the non-remission group according to whether migraine improved one year after the operation. By comparing the preoperative baseline data of the two groups, it was found that there were differences between the two groups in preoperative MIDAS scores, the number of attack days per month before surgery, shunt grading of cTTE, whether there was RLS at rest, CRP, and PCT. When the above indicators were included in the multivariate logistic regression analysis, it was found that preoperative MIDAS scores (*p* = 0.049), number of attack days per month before surgery (*p* = 0.011), the presence of RLS at rest (*p* = 0.049), CRP (*p* = 0.045), and PCT (*p* = 0.010) were independent risk factors for postoperative migraine relief in the metallic occluder group (Tables 4, 5).

**Table 4:** Baseline data between the migraine remission and non-remission groups with metallic occluders.

Variables	Non-remission group *n* = 23	Remission group *n* = 54	*p*-value
Age (Mean ± SD)	41.70 ± 14.24	40.56 ± 12.55	0.727
Sex (*n*, %)			0.825
Female Male	18 (78.3) 5 (21.7)	41 (75.9) 13 (24.1)	
BMI (kg/m^2^)	22.21 ± 3.07	22.60 ± 3.74	0.668
History of hypertension			0.076
Yes No	1 (4.3) 22 (95.7)	11 (20.4) 43 (79.6)	
History of diabetes			0.529
Yes No	1 (4.3) 22 (95.7)	1 (1.9) 53 (98.1)	
History of smoking			0.345
Yes No	1 (4.3) 22 (95.7)	6 (11.1) 48 (88.9)	
LVEF (%)	66.13 ± 5.83	66.04 ± 5.41	0.946
LAD (mm)	30.83 ± 3.73	32.02 ± 4.16	0.240
LVDS (mm)	30.26 ± 3.14	30.15 ± 2.82	0.877
LVDD (mm)	47.17 ± 3.43	47.09 ± 3.72	0.929
cTEE			0.001
Grade II Grade III and above	17 (73.9) 6 (26.1)	17 (31.5) 37 (68.5)	
RLS at rest			0.014
Yes No	1 (4.3) 22 (95.7)	16 (29.6) 38 (70.4)	
DD	0.31 ± 0.19	0.43 ± 0.40	0.282
WBC	6.26 ± 2.15	6.15 ± 1.85	0.823
RBC	4.48 ± 0.60	4.39 ± 0.36	0.451
HB	130.09 ± 19.14	130.46 ± 18.03	0.935
PLT	215.39 ± 52.35	222.37 ± 60.13	0.630
PCT	0.16 ± 0.39	0.22 ± 0.05	<0.001
MPV	9.64 ± 1.04	9.50 ± 0.95	0.561
PDW	10.07 ± 1.07	10.29 ± 1.28	0.675
CRP	2.43 ± 4.18	0.64 ± 0.97	0.001
TG	1.35 ± 0.71	1.56 ± 1.30	0.889
GLU	5.45 ± 0.94	5.35 ± 1.13	0.881
LDL	2.54 ± 0.60	2.68 ± 0.64	0.370
Preoperative MIDAS	32.48 ± 5.40	43.28 ± 6.21	<0.001
Preoperative attack days (/m)	4.52 ± 0.99	6.52 ± 1.81	<0.001

LVEF, left ventricular ejection fraction‌; LAD, left atrial diameter; LVDS, left ventricular end-diastolic volume; LVDD, left ventricular diastolic diameter; cTEE, right heart contrast transthoracic echocardiography; RLS at rest, split right to left in the resting state; DD, D-dimer; WBC, white blood cell count; RBC, red blood cell count; HB, hemoglobin; PLT, platelet count; PCT, platelet hematocrit; MPV, mean platelet volume; PDW, platelet distribution width; CRP, C-reactive protein; TG, triglyceride; GLU, glucose; LDL, low-density lipoprotein.

**Table 5:** Multivariate analysis of the migraine remission and non-remission groups with metallic occluders.

Predictor	Coef	SE	Wald *Z*	*P* value	OR	95%CI
Preoperative MIDAS	0.255	0.130	3.860	0.049	1.291	1.001–1.66
Preoperative attack days	2.528	0.995	6.462	0.011	12.531	1.78–88.007
Shunt grading of cTTE	0.329	1.489	0.049	0.825	1.390	0.075–25.74
RLS at rest	4.971	2.531	3.859	0.049	144.206	1.011–20561.57
PCT	68.487	26.430	6.715	0.010	5.54E+29	17637469.47–1740E+52
CRP	−1.408	0.703	4.008	0.045	0.245	0.062–0.971

RLS at rest, split right to left in the resting state; PCT, platelet hematocrit; CRP, C-reactive protein; Coef, coefficient; SE, standard error; OR, odds ratio; CI, confidence interval.

## Discussion

This study included 133 migraine patients who all had symptoms of varying degrees before the operation. Among them, there were 56 patients with biodegradable PFO occluders and 77 patients with metallic PFO occluders. None of the enrolled patients had serious perioperative complications, and the average operation time and hospital stay were both short. During the operation, TTE can clearly and in real time display the position where the occluder device enters the right atrium from the inferior vena cava and finally reaches the left atrium, as well as its adjacent relationship with the surrounding tissues, providing sufficient guarantee for the safety of the operation. The follow-up at 6/12 months after the operation showed that the RLS of the patients basically disappeared, the MIDAS scores of the two groups significantly decreased, and the headache symptoms were significantly improved. This indicates that the PFO closure in this article can bring significant benefits to migraine patients and has a good therapeutic effect. Compared with metallic occluders, no significant differences were observed in the MIDAS score and the number of migraine attacks each month of patients in the biodegradable occluder group during the 12-month follow-up. It can be seen that the improvement of migraine patients treated with biodegradable occluders can still reach an ideal degree, not inferior to metallic occluders, and there is no long-term metal ion release, tissue compression, etc. The recurrence of migraines in the long term may be lower than that with metallic occluders. Meanwhile, the minor adverse reactions related to the biodegradable occluder group during and after the operation were fewer than those in the metallic occluder group. However, due to the limited relevant data, statistical analysis could not be conducted, and there might be result deviations. Subsequently, more centers and larger sample studies are needed for analysis.

In this study, when comparing the relief factors of migraine between patients in the biodegradable occluder group and the metallic occluder group, it was found that there were no statistically significant differences in gender, age, body mass index, diabetes, hypertension, hyperlipidemia, and cardiac ejection fraction, suggesting that these factors may not be the factors influencing the occurrence of migraine after surgery. As a potential channel, PFO usually does not cause atrial blood shunt under normal circumstances because during the systolic and diastolic periods, the pressure in the left atrium is generally higher. However, when there is a transient increase in the pressure of the right atria, the foramen ovale can open, resulting in RLS at the atrial level. If there are thrombi or microemboli in the venous system or cardiac lumen at this time, they can enter the left cardiac system from the right cardiac system through the patent foramen ovale, causing systemic embolism, that is, paradoxical embolism (21). These emboli of abnormal embolism enter the cerebral arteries through the systemic circulation, causing neurological embolism, which is one of the important causes of cryptogenic stroke (CS) (22). Meanwhile, for some PFO patients with RLS at rest or moderate to large RLS, it is inevitable that blood will be directly shunted from the venous system to the arterial circulation in their daily lives. In this case, various substances (such as vasoactive substances, platelet-activating factors, or amines) are prevented from being filtered and cleared by the pulmonary circulation, thus accumulating in the brain and causing symptoms. Furthermore, microemboli migrate to the distal branches of cerebral arteries through the PFO channel, which may cause cerebral ischemia or inhibition of cortical diffusion, thereby leading to migraine (23). Many studies have shown that approximately 50% of migraine patients with aura are associated with PFO (24–26).

There is still some controversy over the use of PFO closure surgery among migraine patients at present. Currently, no large randomized controlled trials have confirmed the exact efficacy of PFO closure on migraine, although three randomized controlled trials (MIST, PRIMA, and PREMIUM) all reported negative results (19, 27, 28). It was also reported that some patients experienced a decrease in the frequency and intensity of migraine attacks after PFO closure. Meanwhile, multiple single-center studies have shown that PFO closure can effectively control migraine attacks and reduce the intensity, frequency, and duration of migraines (3, 29, 30). The current view holds that, supported by factors such as adequate clinical screening and standardized surgical operations, percutaneous PFO closure can become the preferred treatment strategy for some migraine patients (20, 31–33).

Metallic occluders, as the traditional choice for PFO closure, are facing challenges in terms of their long-term safety. Clinical observations have found that some patients develop new or aggravated migraines after surgery, and the mechanism may be related to metal ion release, persistent endometrial stimulation, and platelet activation. Furthermore, the presence of metallic occluders may prevent related devices from entering the left atrium through the septum in the future treatment of left heart disease (34–36). These phenomena have prompted researchers to explore new blocking strategies that are more in line with the physiological healing process - biodegradable occluders, bring a revolutionary breakthrough to the field of PFO closure. The core advantage of biodegradable occluders (such as MemoSorb®) lies in their biomaterial properties and the design concept of the temporary bridging effect (37). Unlike traditional metallic occluders, it is made of special polylactic acid material. After completing the endothelial tissue bridging task (usually within 6 to 12 months), it gradually degrades into carbon dioxide and water, ultimately achieving an ideal state with no foreign matter residue (38, 39). This process can theoretically avoid metal-related complications. However, whether the degradation and endothelialization rates of biodegradable materials during the material degradation process are matched, whether there are additional inflammatory responses during the degradation process, and their effects on neurovascular functions are not yet clear and require further research to clarify.

Previous related studies have also found that, on the basis of fully selecting patients, there are still some patients whose migraine does not improve significantly after PFO closure. Wang (31) and Zhao (18) respectively conducted multivariate correlation analyses on whether migraine was relieved after metallic occluder implantation and established a nomogram model to predict the predictive factors affecting migraine relief after PFO closure. However, the above studies were all focused on metallic occluders. Then, what are the factors affecting biodegradable occluders, and are there any differences from metallic occluders?

In the two groups of PFO closure, preoperative MIDAS score, the number of attack days per month before surgery, RLS at rest, and PCT were the same independent risk factors for postoperative migraine relief in the two groups of patients, suggesting that for migraine patients with PFO, patients with a higher baseline migraine attack frequency, more severe migraine, RLS at rest, and higher coagulation activity (PCT) are more likely to have postoperative migraine relief. In the metallic occluder group, CRP was an additional independent risk factor for postoperative migraine relief in patients of the metallic occluder group. It is suggested that for patients with metallic devices and lower levels of inflammation (low CRP), postoperative migraines are more likely to be relieved. The influencing factors of the therapeutic effects of the two types of occluders have similarities (higher frequency of migraine attacks, more severe migraine, RLS at rest, and coagulation activity -PCT) and also have differences (the metallic occluder group is also affected by the inflammatory indicator CRP).

As shown in the above results, the MIDAS score is significantly correlated with the frequency and intensity of headache attacks in patients with migraine. A higher MIDAS score and more frequent migraine attacks have a greater impact on patients’ daily life, work, and study. Therefore, no matter which occlusive device is used, patients with higher MIDAS scores and more frequent migraine attacks before the operation can all achieve effective improvement after PFO closure.

Our research found that patients with RLS at rest before the operation could achieve significant improvement in migraine after PFO closure regardless of the type of occluder used. However, the shunt grading of cTTE was only significant for the improvement of migraine in a single factor among the two occluders, while no significant meaning was observed in the multivariate analysis. For this conclusion, we believe that in patients with RLS at rest, the substances in the venous blood that cause migraine attacks are constantly entering the left cardiac artery system through the PFO channel to induce headache attacks. Previous studies have found that the occurrence of migraine may be significantly related to the size of the foramen ovale and the number of shunts, which is consistent with our research (25, 26). Directly closing the PFO channel greatly reduces the entry of the above substances, resulting in a significant improvement of migraine. However, in our study, all patients with foaming grade II or above were selected. For patients with grade II and grade III or above, this grade might not be able to significantly distinguish the amount and quantity of migraine-inducing substances passing through the venous blood during their work and life. Therefore, it might be the reason why the shunt grading of cTTE in this study was not an independent predictor of migraine remission.

This study found that regardless of the type of occluder used, an increase in PCT has predictive value for migraine relief after PFO closure. At present, there is no clear research confirming the correlation between PCT and migraine attacks. PCT is an indicator reflecting the morphology, overall distribution, and proliferation ability of platelets. PCT = platelet count * mean platelet volume/10,000 (40). Previous studies have also found that the platelet/lymphocyte ratio, mean platelet volume, and platelet count in migraine patients are elevated (41). Migraine patients may have higher platelet aggregation due to the influence of various vasoactive substances (42, 43). PCT may represent platelet aggregation. Therefore, reducing the transmission of vasoactive substances after PFO closure improves migraine attacks. However, the mechanism of PCT and migraine relief after PFO closure still needs further research to be clarified.

For migraine patients with a high level of CRP, it may itself indicate that there is a highly non-specific systemic inflammatory response during the onset of migraine in this patient (44). Compared with metallic occluders, biodegradable occluders do not have inflammatory reactions caused by the release of metal ions or inflammation caused by the continuous compression and wear of surrounding tissues. Therefore, the level of CRP has no predictive significance for the improvement of migraine after the implantation of biodegradable occluders. However, a lower level of CRP reduces the probability of systemic non-specific inflammatory responses after the implantation of metallic occluders, resulting in the improvement of migraine.

In this study, there are still some limitations. Firstly, the sample size included in this study is small, and the follow-up time is short. Therefore, it is impossible to clarify the long-term effect results of PFO closure on migraine, especially the effect results on migraine after absorption by biodegradable occluders. Secondly, in this study, there were relatively few high-risk and complex abnormal foramen ovale structures. Therefore, the interventional closure rate of PFO in the patients included in this study was high, and there were few complications. It may not be extended to all PFO patients because the success of PFO closure is related to the anatomical complexity of PFO and whether PFO is high-risk. Meanwhile, since some patients refuse to be followed up during the postoperative follow-up process, it may cause bias in the results. For example, patients with good symptom relief are even less willing to have a re-examination. Thirdly, patients after PFO closure need to take antiplatelet drugs orally for varying durations. Therefore, the potential interference of antiplatelet drug treatment on the severity of migraine cannot be completely ruled out. This study did not incorporate a drug treatment control group, which may limit the generalizability of the findings. Our team will continue to follow up on the above-mentioned deficiencies and increase the sample size in response to the problem of small sample size.

## Conclusion

Our research found that PFO closure is an effective option for some migraine patients (especially those with severe migraine symptoms or RLS at rest); biodegradable occluders offer comparable short-term migraine symptom improvement effects to metallic occluders and may have better long-term safety prospects. Preoperative assessment of the severity of migraine, shunt characteristics (RLS at rest), coagulation function (PCT), and inflammatory status (CRP, especially when using metallic occluders) is helpful for predicting the possibility of postoperative remission. Under the premise of comparable therapeutic effects, biodegradable occluders may become a better choice for specific populations (such as young patients and those allergic to nickel) due to their better biocompatibility and long-term safety. The sample size of this study is small, and the follow-up period is short, which may lead to bias in the outcome. Instead, it can be considered an excellent starting point for a well-designed prospective multicenter. It is necessary to carry out larger-scale, longer-follow-up-period, prospective, especially stratified-design-by-migraine-type RCTs in the future to confirm the efficacy and advantages of biodegradable occluders.

## Data Availability

The original contributions presented in the study are included in the article/supplementary material, further inquiries can be directed to the corresponding author/s.

## References

[1] Hara H Virmani R Ladich E Mackey-Bojack S Titus J Reisman M. Patent foramen ovale: current pathology, pathophysiology, and clinical status. J Am Coll Cardiol. (2005) 46:1768–76. doi: 10.1016/j.jacc.2005.08.038, PMID: 16256883

[2] Collado FMS Poulin MF Murphy JJ Jneid H Kavinsky CJ. Patent foramen Ovale closure for stroke prevention and other disorders. J Am Heart Assoc. (2018) 7:e007146. doi: 10.1161/JAHA.117.007146, PMID: 29910192 PMC6220531

[3] Evola S Camarda EA Triolo OF Adorno D D'Agostino A Novo G. Clinical outcomes and quality of life after patent foramen ovale (PFO) closure in patients with stroke/transient ischemic attack of undetermined cause and other PFO-associated clinical conditions: a single-center experience. J Clin Med. (2023) 12:5788. doi: 10.3390/jcm12185788, PMID: 37762729 PMC10531865

[4] Qi Y Zhang Y Luo X Cheng G Du Y Liu R. Efficacy of patent foramen ovale closure for treating migraine: a prospective follow-up study. J Investig Med. (2021) 69:7–12. doi: 10.1136/jim-2020-001323, PMID: 32928904 PMC7803887

[5] Antnio C Silveira M Maria José JMD Castillo. Point of View Echocardiographic Evaluation of Patients with Patent Foramen Ovale and Cryptogenic Stroke[J]. Arq Bras Cardiol: Imagem Cardiovasc (2021) 34:eabc123. doi: 10.47593/2675-312X/20213401eabc123

[6] Mas JL Guillon B Charles-Nelson A Domigo V Derex L Massardier E. Patent foramen ovale closure in stroke patients with migraine in the CLOSE trial. The CLOSE-MIG study. Eur J Neurol. (2021) 28:2700–7. doi: 10.1111/ene.14892, PMID: 33938088

[7] Vetvik KG MacGregor EA. Sex differences in the epidemiology, clinical features, and pathophysiology of migraine. Lancet Neurol. (2017) 16:76–87. doi: 10.1016/S1474-4422(16)30293-9, PMID: 27836433

[8] Safiri S Pourfathi H Eagan A Mansournia MA Khodayari MT Sullman MJM. Global, regional, and national burden of migraine in 204 countries and territories, 1990 to 2019. Pain. (2022) 163:e293–e309. doi: 10.1097/j.pain.0000000000002275 34001771

[9] Takizawa T Ayata C Chen SP. Therapeutic implications of cortical spreading depression models in migraine. Prog Brain Res. (2020) 255:29–67. doi: 10.1016/bs.pbr.2020.05.009 33008510

[10] Wilmshurst PT Nightingale S Walsh KP Morrison WL. Effect on migraine of closure of cardiac right-to-left shunts to prevent recurrence of decompression illness or stroke or for haemodynamic reasons. Lancet. (2000) 356:1648–51. doi: 10.1016/S0140-6736(00)03160-3, PMID: 11089825

[11] Liu K Wang BZ Hao Y Song S Pan M. The correlation between migraine and patent foramen ovale. Front Neurol. (2020) 11:543485. doi: 10.3389/fneur.2020.543485, PMID: 33335507 PMC7736411

[12] Sharma V DeShazo RA Skidmore CR Glotzbach JP Koliopoulou A Javan H. Surgical explantation of atrial septal closure devices for refractory nickel allergy symptoms. J Thorac Cardiovasc Surg. (2020) 160:502–9.e1. doi: 10.1016/j.jtcvs.2019.10.177 31959452

[13] Tsinivizov P Giannakopoulos A Varvarousis D Michelinakis N Koumallos N Manolis AJ. Cardiac tamponade due to very late perforation of left atrium by atrial septal defect Occluder. JACC Cardiovasc Interv. (2021) 14:e49–51. doi: 10.1016/j.jcin.2020.11.039, PMID: 33582082

[14] Bahrani B Moghaddam N DeKoven J. Cross-sectional survey of nickel allergy Management in the Context of Intracardiac device implantation. Dermatitis. (2019) 30:213–21. doi: 10.1097/DER.0000000000000466, PMID: 31045931

[15] Chessa M Carminati M Butera G Bini RM Drago M Rosti L. Early and late complications associated with transcatheter occlusion of secundum atrial septal defect. J Am Coll Cardiol. (2002) 39:1061–5. doi: 10.1016/S0735-1097(02)01711-4, PMID: 11897451

[16] Carminati M Butera G Chessa M De Giovanni J Fisher G Gewillig M. Transcatheter closure of congenital ventricular septal defects: results of the European registry. Eur Heart J. (2007) 28:2361–8. doi: 10.1093/eurheartj/ehm314, PMID: 17684082

[17] Mas JL Derumeaux G Guillon B Massardier E Hosseini H Mechtouff L. Patent foramen ovale closure or anticoagulation vs. antiplatelets after stroke. N Engl J Med. (2017) 377:1011–21. doi: 10.1056/NEJMoa1705915, PMID: 28902593

[18] Zhao E Xie H Zhang Y. A nomogram for the prediction of cessation of migraine among patients with patent foramen ovale after percutaneous closure. Front Neurol. (2020) 11:593074. doi: 10.3389/fneur.2020.593074, PMID: 33193059 PMC7645229

[19] Tobis JM Charles A Silberstein SD Sorensen S Maini B Horwitz PA. Percutaneous closure of patent foramen ovale in patients with migraine: the PREMIUM trial. J Am Coll Cardiol. (2017) 70:2766–74. doi: 10.1016/j.jacc.2017.09.1105, PMID: 29191325

[20] Ben-Assa E Rengifo-Moreno P Al-Bawardy R Kolte D Cigarroa R Cruz-Gonzalez I. Effect of residual interatrial shunt on migraine burden after Transcatheter closure of patent foramen Ovale. JACC Cardiovasc Interv. (2020) 13:293–302. doi: 10.1016/j.jcin.2019.09.042, PMID: 32029246

[21] Windecker S Stortecky S Meier B. Paradoxical embolism. J Am Coll Cardiol. (2014) 64:403–15. doi: 10.1016/j.jacc.2014.04.063, PMID: 25060377

[22] Wessler BS Kent DM. Prevention of recurrent stroke in patients with patent foramen ovale. Neurol Clin. (2015) 33:491–500. doi: 10.1016/j.ncl.2015.01.002, PMID: 25907918 PMC4409665

[23] Finocchi C Del Sette M. Migraine with aura and patent foramen ovale: myth or reality? Neurol Sci. (2015) 36:61–6. doi: 10.1007/s10072-015-2163-8 26017514

[24] Kumar P Kijima Y West BH Tobis JM. The connection between patent foramen Ovale and migraine. Neuroimaging Clin N Am. (2019) 29:261–70. doi: 10.1016/j.nic.2019.01.006, PMID: 30926116

[25] Kijima Y Miller N Noureddin N Gevorgyan R Tobis J. TCT-738 the degree of right-to-left shunt is associated with visual aura due to migraine [J]. Journal of the American College of Cardiology (2015) 66:B301-B. doi: 10.1016/j.jacc.2015.08.761

[26] Giblett JP Abdul-Samad O Shapiro LM Rana BS Calvert PA. Patent foramen ovale closure in 2019. Interventional Cardiol. (2019) 14:34–41. doi: 10.15420/icr.2018.33.2, PMID: 30858890 PMC6406129

[27] Dowson A Mullen MJ Peatfield R Muir K Khan AA Wells C. Migraine intervention with STARFlex technology (MIST) trial: a prospective, multicenter, double-blind, sham-controlled trial to evaluate the effectiveness of patent foramen ovale closure with STARFlex septal repair implant to resolve refractory migraine headache. Circulation. (2008) 117:1397–404. doi: 10.1161/CIRCULATIONAHA.107.727271, PMID: 18316488

[28] Mattle HP Evers S Hildick-Smith D Becker WJ Baumgartner H Chataway J. Percutaneous closure of patent foramen ovale in migraine with aura, a randomized controlled trial. Eur Heart J. (2016) 37:2029–36. doi: 10.1093/eurheartj/ehw027, PMID: 26908949

[29] Rigatelli G Dell'Avvocata F Ronco F Cardaioli P Giordan M Braggion G. Primary transcatheter patent foramen ovale closure is effective in improving migraine in patients with high-risk anatomic and functional characteristics for paradoxical embolism. JACC Cardiovasc Interv. (2010) 3:282–7. doi: 10.1016/j.jcin.2009.11.019, PMID: 20298985

[30] Wahl A Praz F Tai T Findling O Walpoth N Nedeltchev K. Improvement of migraine headaches after percutaneous closure of patent foramen ovale for secondary prevention of paradoxical embolism. Heart. (2010) 96:967–73. doi: 10.1136/hrt.2009.181156, PMID: 20538672

[31] Wang Y Lou Y Chen Y Shi J Zhang H. Construction and validation of a nomogram for predicting remission of migraine patients with patent foramen ovale after closure. Int J Cardiol. (2024) 407:132026. doi: 10.1016/j.ijcard.2024.132026, PMID: 38609055

[32] Xing YQ Guo YZ Gao YS Guo ZN Niu PP Yang Y. Effectiveness and safety of Transcatheter patent foramen Ovale closure for migraine (EASTFORM) trial. Sci Rep. (2016) 6:39081. doi: 10.1038/srep39081, PMID: 27966652 PMC5155423

[33] He YD Yan XL Qin C Zhang P Guo ZN Yang Y. Transcatheter patent foramen Ovale closure is effective in alleviating migraine in a 5-year follow-up. Front Neurol. (2019) 10:1224. doi: 10.3389/fneur.2019.01224, PMID: 31803135 PMC6877730

[34] Krumsdorf U Ostermayer S Billinger K Trepels T Zadan E Horvath K. Incidence and clinical course of thrombus formation on atrial septal defect and patient foramen ovale closure devices in 1,000 consecutive patients. J Am Coll Cardiol. (2004) 43:302–9. doi: 10.1016/j.jacc.2003.10.030, PMID: 14736453

[35] Iskander B Anwer F Oliveri F Fotios K Panday P Arcia Franchini AP. Amplatzer patent foramen Ovale Occluder device-related complications. Cureus. (2022) 14:e23756. doi: 10.7759/cureus.23756, PMID: 35402119 PMC8980243

[36] Kashyap T Sanusi M Momin ES Khan AA Mannan V Pervaiz MA. Transcatheter Occluder devices for the closure of atrial septal defect in children: how safe and effective are they? A systematic review. Cureus. (2022) 14:e25402. doi: 10.7759/cureus.25402, PMID: 35765405 PMC9233908

[37] Guo G Hu J Wang F Fu D Luo R Zhang F. A fully degradable transcatheter ventricular septal defect occluder: Towards rapid occlusion and post-regeneration absorption. Biomaterials. (2022) 291:121909. doi: 10.1016/j.biomaterials.2022.121909 36401954

[38] Ali SA Zhong SP Doherty PJ Williams DF. Mechanisms of polymer degradation in implantable devices. I. Poly(caprolactone). Biomaterials. (1993) 14:648–56. doi: 10.1016/0142-9612(93)90063-8 8399961

[39] Tsuji H Ikarashi K. In vitro hydrolysis of poly(L-lactide) crystalline residues as extended-chain crystallites. Part I: long-term hydrolysis in phosphate-buffered solution at 37 degrees C. Biomaterials. (2004) 25:5449–55. doi: 10.1016/j.biomaterials.2003.12.053 15142725

[40] Budak YU Polat M Huysal K. The use of platelet indices, plateletcrit, mean platelet volume and platelet distribution width in emergency non-traumatic abdominal surgery: a systematic review. Biochem Med. (2016) 26:178–93. doi: 10.11613/BM.2016.020, PMID: 27346963 PMC4910273

[41] Sarıcam G. Relationship between migraine headache and hematological parameters. Acta Neurol Belg. (2021) 121:899–905. doi: 10.1007/s13760-020-01362-x, PMID: 32347450

[42] Borgdorff P Tangelder GJ. Migraine: possible role of shear-induced platelet aggregation with serotonin release [J]. Headache, (2012) 52:1298–318. doi: 10.1111/j.1526-4610.2012.02162.x 22568554

[43] Danese E Montagnana M Lippi G. Platelets and migraine. Thromb Res. (2014) 134:17–22. doi: 10.1016/j.thromres.2014.03.055, PMID: 24767953

[44] Wang Y Wang Y Zhang J Jiang C. Association of 5-HT, ET-1, PTX-3, and inflammatory markers with clinical parameters in pediatric migraine patients with patent foramen ovale. Eur J Paediatric Neurol. (2025) 58:14–9. doi: 10.1016/j.ejpn.2025.07.003, PMID: 40683192

